# FLT3 Mutations in Acute Myeloid Leukemia: Unraveling the Molecular Mechanisms and Implications for Targeted Therapies

**DOI:** 10.7759/cureus.45765

**Published:** 2023-09-22

**Authors:** Meryem Jalte, Meriame Abbassi, Hinde El Mouhi, Hanae Daha Belghiti, Mohamed Ahakoud, Hicham Bekkari

**Affiliations:** 1 Laboratory of Biotechnology, Environment, Agri-Food, and Health (LBEAH), Faculty of Sciences Dhar El Mahraz, Sidi Mohamed Ben Abdellah University, Fez, MAR; 2 Laboratory of Biomedical and Translational Research, Faculty of Medicine and Pharmacy and Dental Medicine, Sidi Mohamed Ben Abdellah University, Fez, MAR; 3 Laboratory of Medical Genetics and Oncogenetics, Hospital University Hassan II, Fez, MAR

**Keywords:** acute myeloid leukemia (aml), flt3 mutations, flt3 signaling, targeted therapies, therapeutic resistance

## Abstract

Acute myeloid leukemia (AML) is a heterogeneous and aggressive form of blood cancer characterized by the uncontrolled proliferation of myeloid precursor cells in the bone marrow. It affects individuals of all ages, with incidence increasing notably in those over 65 years old. Despite advancements in treatment, overall survival rates remain unsatisfactory, underscoring the need for a deeper understanding of the disease. Among the various genetic alterations implicated in AML pathogenesis, mutations in the *FLT3* (Fms-like tyrosine kinase 3) gene have emerged as significant contributors to leukemogenesis. The *FLT3* ​​​​​gene encodes a type III receptor tyrosine kinase crucial in regulating normal hematopoiesis. Approximately one-third of AML patients carry *FLT3* mutations, making it one of the most frequently mutated genes in the disease. *FLT3 *mutations can be classified into internal tandem duplications (ITDs) and point mutations in the tyrosine kinase domain (TKD). *FLT3* mutations are associated with adverse clinical features and are independent prognostic factors for poor overall survival and decreased remission rates in AML patients. Understanding the molecular mechanisms underlying *FLT3* mutations in AML is critical for improving risk stratification, prognosis assessment, and the development of targeted therapies. By reviewing the current literature, this study aims to elucidate the functional consequences of *FLT3* mutations in AML pathogenesis, explore the interaction of *FLT3* signaling with other oncogenic pathways, and assess the prognostic significance of *FLT3* mutations in clinical practice, providing information that can guide future research directions and facilitate the development of more effective therapeutic strategies.

## Introduction and background

Background of acute myeloid leukemia (AML)

AML represents a heterogeneous and aggressive hematologic malignancy characterized by the uncontrolled proliferation of myeloid precursor cells within the bone marrow. Despite therapeutic advancements, AML continues to exhibit a dismal prognosis, underscoring the imperative for enhanced comprehension of its pathogenesis and treatment modalities [1].

Importance of *FLT3* mutations in AML

Among the various genetic alterations implicated in AML pathogenesis, mutations in the *FLT3* (Fms-like tyrosine kinase 3) gene have emerged as a significant contributor to leukemogenesis. The *FLT3* gene encodes a receptor tyrosine kinase that plays a crucial role in regulating normal hematopoiesis, including the proliferation, survival, and differentiation of hematopoietic stem and progenitor cells [2]. Approximately one-third of AML patients carry *FLT3* mutations, making it one of the most frequently mutated genes in this disease [3]. *FLT3* mutations can be broadly classified into two main types: internal tandem duplications (ITDs) and point mutations within the tyrosine kinase domain (TKD) [4]. These mutations result in constitutive activation of *FLT3* signaling, leading to aberrant proliferation, impaired differentiation, and increased survival of leukemic cells. *FLT3* mutations are associated with adverse clinical features, including higher white blood cell counts, increased blast percentage, and a higher likelihood of relapse [5]. They have also been identified as independent prognostic factors for poor overall survival and decreased remission rates in AML patients [6]. Therefore, understanding the molecular mechanisms underlying *FLT3*-mutated AML is crucial for improving risk stratification, prognosis assessment, and development of targeted therapies.

Purpose of the research paper

The purpose of this research paper is to provide a comprehensive analysis of *FLT3* mutations in AML, focusing on their molecular mechanisms and implications for targeted therapies. By reviewing the current literature, we aim to elucidate the functional consequences of *FLT3* mutations in AML pathogenesis, explore the interaction of *FLT3* signaling with other oncogenic pathways, and evaluate the prognostic significance of *FLT3* mutations in clinical practice. Furthermore, we will discuss the recent advances in targeted therapies against *FLT3*-mutated AML, such as first- and second-generation *FLT3* inhibitors, and highlight the challenges associated with their use, including the development of resistance mechanisms. Additionally, we will explore emerging therapeutic strategies, including third-generation inhibitors and combination approaches, as well as the potential role of immunotherapy in the management of *FLT3*-mutated AML.

By examining the latest research findings and discussing the clinical implications, this paper aims to contribute to a better understanding of *FLT3 *mutations in AML and provide insights that may guide future research directions and facilitate the development of more effective therapeutic strategies. Moreover, a recent study conducted in 2021 revealed the clonal evolution of AML with *FLT3*-ITD mutation under treatment with midostaurin, showcasing the dynamic nature of *FLT3* mutations and their potential implications for treatment response [7]. The findings of this study underscore the importance of gaining a deeper understanding of *FLT3* mutations and their influence on treatment outcomes, further highlighting the significance of investigating *FLT3* mutations in AML.

## Review

*FLT3* gene and its role in hematopoiesis

Overview of the FLT3 Gene

**Figure 1:**
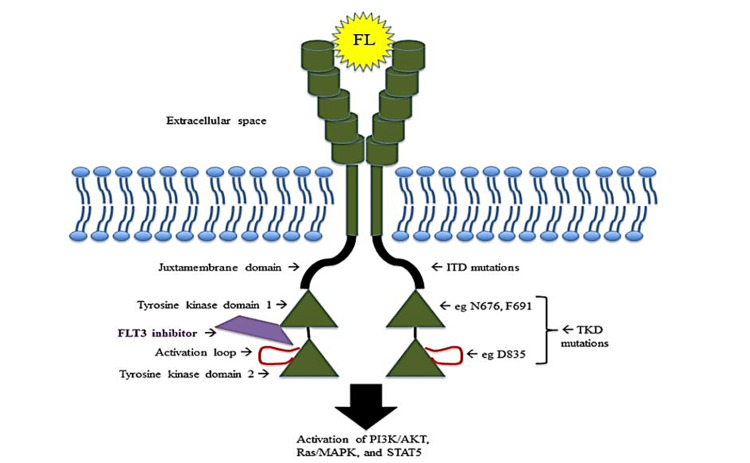
Schematic representation of FLT3 structure and mutations. Schematic representation of *FLT3* structure and the location of *FLT3*-ITD and *FLT3*-TKD mutations within the receptor. *FLT3*-ITD mutations result in tandem duplications in the juxtamembrane domain, while *FLT3*-TKD mutations involve substitutions in the TKD. Source: [10]. FLT3, Fms-like tyrosine kinase 3; FL, FLT3 ligand; TKD, tyrosine kinase domain; ITD, internal tandem duplication

The *FLT3* gene, also known as CD135, is situated on chromosome 13q12 and encodes a type III receptor tyrosine kinase. Comprising 24 exons, the *FLT3* gene undergoes alternative splicing, giving rise to several isoforms. Notably, the primary isoform, *FLT3*-ITD, frequently associated with AML, is distinguished by the insertion of tandem duplications of varying lengths within the gene's coding region, particularly within the juxtamembrane domain [8,9]. This genetic alteration at the DNA level has significant implications for the function and activation of the encoded protein, influencing its role in AML pathogenesis [8,9]. To better understand the structural alterations caused by *FLT3* mutations, Figure 1 provides a schematic representation of *FLT3 *and highlights the positions of *FLT3*-ITD and *FLT3*-TKD mutations within the receptor. *FLT3*-ITD mutations involve tandem duplications in the juxtamembrane domain, while *FLT3*-TKD mutations result in substitutions in the TKD.

Normal Function of FLT3 in Hematopoiesis

In normal hematopoiesis, *FLT3* plays a pivotal role in the development and maintenance of hematopoietic stem and progenitor cells (HSPCs). Binding of its ligand, *FLT3* ligand (FL), leads to dimerization and autophosphorylation of the *FLT3* receptor, initiating downstream signaling cascades [11]. Activation of *FLT3* promotes the survival, proliferation, and differentiation of HSPCs, thereby regulating the production of mature blood cells [12].

Signaling Pathways Activated by FLT3

Upon activation, *FLT3* triggers several signaling pathways that contribute to its role in hematopoiesis. One of the key pathways is the RAS/mitogen-activated protein kinase (MAPK) pathway, which regulates cell proliferation and survival [13]. *FLT3* activation also leads to the activation of phosphatidylinositol 3-kinase (PI3K)/AKT signaling, which promotes cell survival and inhibits apoptosis [14]. Additionally, the signal transducer and activator of transcription (STAT) pathway are activated downstream of *FLT3*, playing a role in cell survival, differentiation, and proliferation [15].

Importance of FLT3 Mutations in Disturbing Hematopoiesis

*FLT3* mutations, particularly ITDs and TKD mutations, have a profound impact on hematopoiesis. *FLT3*-ITD mutations lead to the constitutive activation of *FLT3* signaling, resulting in dysregulated proliferation, impaired differentiation, and enhanced survival of leukemic cells [11]. These mutations disrupt the balance of hematopoietic cell populations, leading to the expansion of undifferentiated blasts and the inhibition of normal hematopoietic cell production. *FLT3* mutations contribute to leukemogenesis by conferring a growth advantage to leukemic cells, enabling them to bypass normal regulatory mechanisms and promote the expansion of malignant clones. The altered signaling pathways downstream of mutated *FLT3* dysregulate key cellular processes, disrupting the normal hematopoietic hierarchy and contributing to the development of AML [15]. The presence of *FLT3* mutations, particularly *FLT3*-ITD, is associated with adverse clinical features, such as higher leukocyte counts, increased risk of relapse, and reduced overall survival in AML patients [8]. Understanding the impact of *FLT3* mutations on hematopoiesis is essential for unraveling the mechanisms underlying leukemogenesis and developing targeted therapies that specifically inhibit aberrant *FLT3* signaling to restore normal hematopoiesis.

Types and prevalence of *FLT3* mutations in AML

The types and prevalence of *FLT3* mutations in AML are summarized in Table 1. *FLT3*-ITD mutations, occurring in the juxtamembrane domain and resulting in tandem duplications, are the most common *FLT3 *mutation, accounting for approximately 25% to 30% of AML cases [8]. Point mutations in the TKD of *FLT3* are less common, occurring in approximately 5% to 10% of AML cases [9].

**Table 1:** Types and prevalence of FLT3 mutations in AML. FLT3, Fms-like tyrosine kinase 3; TKD, tyrosine kinase domain; ITD, internal tandem duplication; AML, acute myeloid leukemia

Mutation type	Definition and prevalence
ITD	*FLT3*-ITD mutations occur in the juxtamembrane domain and result in the insertion of tandem duplications of variable lengths. They are the most common *FLT3* mutation in AML, accounting for approximately 25%-30% of cases.
Point mutations (TKD mutations)	*FLT3*-TKD mutations involve substitutions within the TKD of *FLT3*. They are less common than *FLT3*-ITD mutations and occur in approximately 5%-10% of AML cases.

ITD Mutations

Definition and prevalence: ITD mutations in the *FLT3* gene involve the insertion of tandem duplications of varying lengths within the juxtamembrane domain. These insertions result in a constitutively activated *FLT3* receptor and dysregulated signaling [2]. ITD mutations are the most common *FLT3* mutations in AML, accounting for approximately 20% to 30% of cases [16].

Impact on the *FLT3* function: *FLT3*-ITD mutations lead to ligand-independent activation of the receptor and increased autophosphorylation, leading to the activation of downstream signaling pathways. This results in aberrant cell proliferation, impaired differentiation, and enhanced cell survival [2]. The length and location of ITD mutations may influence their impact on *FLT3* signaling and clinical outcomes.

Prognostic significance in AML patients: AML patients with *FLT3*-ITD mutations often present with adverse clinical features, including higher leukocyte counts, increased blast percentage, and a higher likelihood of relapse [6]. *FLT3*-ITD mutations are associated with a decreased complete remission rate and poor overall survival, particularly in patients with a high allelic ratio or the presence of additional genetic abnormalities [17]. Furthermore, *FLT3*-ITD status has been incorporated into risk stratification systems for AML, aiding in treatment decisions and prognosis assessment.

Furthermore, it has been reported that specific *FLT3* D835/I836 mutations are associated with poor disease-free survival and a distinct gene expression signature in younger adults with de novo cytogenetically normal AML lacking *FLT3* ITDs [2]. This finding highlights the relevance of considering different *FLT3* mutation types and their impact on AML outcomes [18].

Point Mutations (TKD Mutations)

Definition and prevalence: Point mutations in the TKD of the *FLT3* gene involve single amino acid substitutions within critical regions of the receptor. These mutations confer constitutive *FLT3* activation and are distinct from ITD mutations in terms of their molecular mechanisms and clinical implications. TKD mutations occur in approximately 7% to 10% of AML cases [4].

Consequences for *FLT3* activity and clinical outcomes: *FLT3*-TKD mutations result in an increased kinase activity of the receptor, leading to constitutive activation of downstream signaling pathways [2]. Although TKD mutations are generally associated with a milder phenotype compared to *FLT3*-ITD mutations, their prognostic impact in AML remains controversial. Some studies suggest an adverse effect on clinical outcomes, such as decreased overall survival and increased relapse rates [8], while others report no significant association with prognosis [17]. Further investigations are needed to clarify the prognostic relevance of *FLT3*-TKD mutations.

Co-occurrence of FLT3 Mutations With Other Genetic Abnormalities in AML

*FLT3* mutations often co-occur with other genetic abnormalities in AML, contributing to disease heterogeneity and influencing clinical outcomes. Common co-occurring genetic alterations include NPM1 mutations, DNMT3A mutations, and mutations in genes involved in chromatin modification (such as ASXL1 and IDH1/2) [8]. The presence of FLT3 mutations, particularly *FLT3*-ITD, in combination with other genetic abnormalities, may confer additional adverse prognostic implications and impact treatment strategies.

Understanding the prevalence and implications of different *FLT3* mutations in AML is essential for risk stratification, treatment decisions, and the development of targeted therapies aimed at specifically inhibiting *FLT3* signaling pathways.

Molecular mechanisms of *FLT3* mutations in AML pathogenesis

Constitutive Activation of FLT3 Signaling Pathways

Impact on cell proliferation and survival: *FLT3* mutations result in the constitutive activation of *FLT3* signaling pathways, leading to dysregulated cell proliferation and enhanced cell survival in AML. The aberrant activation of *FLT3* signaling promotes the proliferation of leukemic cells by driving cell cycle progression and overcoming normal growth regulatory mechanisms [19]. This uncontrolled cell proliferation contributes to the expansion of leukemic blasts in the bone marrow and peripheral blood.

Moreover, *FLT3* mutations confer a survival advantage to leukemic cells by inhibiting apoptosis, the programmed cell death process. Constitutively active *FLT3* signaling activates anti-apoptotic pathways, such as the PI3K/AKT and RAS/MAPK pathways, leading to the suppression of cell death signals [20]. Consequently, leukemic cells with *FLT3* mutations exhibit increased resistance to apoptosis, contributing to their prolonged survival.

Influence on differentiation and self-renewal of hematopoietic stem/progenitor cells: *FLT3* mutations also disrupt the normal differentiation and self-renewal processes of HSPCs. In normal hematopoiesis, *FLT3* signaling plays a crucial role in regulating the balance between self-renewal and differentiation of HSPCs [12]. However, *FLT3* mutations disturb this balance, promoting the expansion of undifferentiated progenitor cells at the expense of mature blood cell production.

*FLT3*-ITD mutations, in particular, have been associated with a blockage of myeloid differentiation, leading to the accumulation of undifferentiated myeloblasts in AML [2]. This differentiation block contributes to the aggressive phenotype of *FLT3*-ITD AML, as immature blasts are less responsive to differentiation-inducing agents and are associated with poor treatment outcomes.

Interaction With Other Oncogenic Pathways

Crosstalk with RAS and JAK-STAT signaling: *FLT3* signaling pathways interact and crosstalk with other oncogenic pathways, amplifying the leukemogenic effects of *FLT3* mutations. One important interaction is between *FLT3* and the RAS pathway. Activated *FLT3* can stimulate RAS signaling, leading to further enhancement of cell proliferation and survival [15]. This crosstalk between *FLT3* and RAS pathways contributes to the aggressive nature of *FLT3*-mutated AML.

Additionally, *FLT3* mutations can activate the JAK-STAT signaling pathway. Activation of STAT proteins downstream of *FLT3* promotes cell survival, proliferation, and resistance to apoptosis [13]. The interplay between *FLT3* and JAK-STAT signaling pathways further supports the leukemogenic potential of *FLT3* mutations.

Synergistic effects on leukemogenesis: *FLT3* mutations can also act synergistically with other genetic abnormalities in AML, leading to an increased leukemogenic potential. Co-occurrence of *FLT3* mutations with mutations in genes such as NPM1, DNMT3A, or IDH1/2 can have additive or synergistic effects on leukemogenesis [2]. These interactions may involve shared downstream signaling pathways or cooperativity in disrupting normal hematopoietic processes.

Microenvironmental Factors and FLT3 Mutations

The microenvironment of the bone marrow plays a critical role in AML pathogenesis, and *FLT3* mutations can influence the interactions between leukemic cells and their surrounding niche. The bone marrow microenvironment provides signals that support the survival and growth of leukemic cells, and *FLT3* mutations can modulate the responsiveness of leukemic cells to these signals [14]. Additionally, factors such as cytokines, stromal cells, and extracellular matrix components in the microenvironment can further activate *FLT3* signaling or contribute to drug resistance in *FLT3*-mutated AML.

Understanding the molecular mechanisms underlying *FLT3* mutations in AML pathogenesis is essential for developing targeted therapies and improving treatment outcomes for patients with *FLT3*-mutated AML.

**Figure 2:**
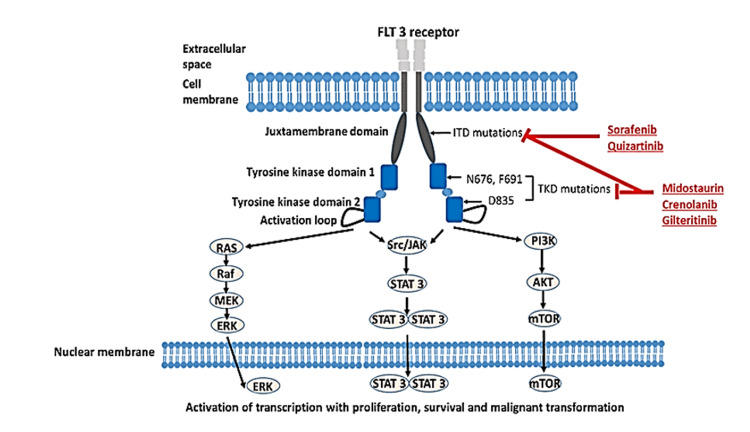
FLT3 Signaling pathways and their dysregulation in AML. Illustration of normal *FLT3* signaling pathways involved in hematopoiesis and the dysregulated signaling pathways caused by *FLT3* mutations in AML. *FLT3* mutations lead to constitutive activation of downstream signaling pathways, promoting cell proliferation, survival, and impaired differentiation. Source: [21]. FLT3, Fms-like tyrosine kinase 3; TKD, tyrosine kinase domain; ITD, internal tandem duplication; STAT, signal transducer and activator of transcription; mTOR, mammalian target of rapamycin; ERK, extracellular signal-regulated kinase; PI3K, phosphoinositide 3-kinase

To elucidate the molecular mechanisms underlying the pathogenesis of AML, Figure 2 depicts the normal *FLT3* signaling pathways involved in hematopoiesis and the dysregulated signaling pathways caused by *FLT3* mutations in AML. The figure illustrates how *FLT3* mutations result in constitutive activation of downstream signaling pathways, leading to aberrant cell proliferation, enhanced survival, and impaired differentiation.

Clinical implications and targeted therapies

Prognostic Value of FLT3 Mutations in AML

Impact on overall survival and relapse rates: *FLT3* mutations have significant prognostic implications in AML patients. Several studies have consistently shown that AML patients with *FLT3*-ITD mutations have poorer overall survival compared to those without *FLT3* mutations [6]. *FLT3*-ITD mutations are associated with higher relapse rates, shorter duration of remission, and increased risk of treatment failure [17]. The presence of *FLT3*-ITD mutations, particularly with high allelic ratio and additional adverse cytogenetic abnormalities, often indicates a more aggressive disease course.

Risk stratification in clinical practice: Due to the prognostic significance of *FLT3* mutations, they are incorporated into risk stratification systems to guide treatment decisions in AML. The European LeukemiaNet (ELN) and other guidelines consider *FLT3*-ITD mutations as an adverse risk factor and classify patients accordingly [6]. Risk stratification helps identify patients who may benefit from more intensive therapies or targeted agents.

Overview of FLT3 Inhibitors

First-generation inhibitor midostaurin: Midostaurin is a multi-targeted kinase inhibitor that was the first *FLT3* inhibitor approved by regulatory authorities for the treatment of *FLT3*-mutated AML. It inhibits both *FLT3*-ITD and *FLT3*-TKD mutations, as well as other kinases. Clinical trials have shown that the addition of midostaurin to standard chemotherapy improves overall survival and increases the rate of complete remission in newly diagnosed *FLT3-*mutated AML patients [22]. Midostaurin is typically used in combination with chemotherapy and has become a standard of care for this patient population.

Second-generation inhibitors gilteritinib and quizartinib: Second-generation *FLT3* inhibitors, such as gilteritinib and quizartinib, have been developed to overcome the limitations of first-generation inhibitors. These agents specifically target *FLT3* and exhibit higher potency and selectivity for *FLT3* mutations. Gilteritinib and quizartinib have shown promising clinical activity as single agents in relapsed or refractory *FLT3*-mutated AML patients [23]. They have demonstrated high response rates and significant improvements in overall survival in this patient population. Ongoing studies are evaluating their efficacy in combination with chemotherapy or other targeted agents.

Challenges and Limitations in FLT3-Targeted Therapy

Resistance mechanisms and relapse: Despite the initial responses observed with *FLT3* inhibitors, the development of resistance remains a significant challenge in *FLT3*-mutated AML. Resistance mechanisms include the acquisition of secondary *FLT3* mutations, activation of alternative signaling pathways, and alterations in the bone marrow microenvironment [24]. These resistance mechanisms can lead to relapse or incomplete response to targeted therapy.

Combination strategies and future directions: To address the challenge of resistance and improve outcomes in *FLT3*-mutated AML, combination strategies are being explored. Combinations of *FLT3* inhibitors with chemotherapy, other targeted agents, or immune-based therapies are being investigated in clinical trials [20]. Additionally, novel *FLT3* inhibitors with enhanced potency and selectivity are being developed to overcome resistance and improve treatment outcomes. Furthermore, the identification of additional molecular targets and understanding of the complex interplay between signaling pathways hold promise for future therapeutic interventions in *FLT3*-mutated AML.

*FLT3* mutations in AML have important clinical implications, affecting prognosis, risk stratification, and treatment decisions. *FLT3* inhibitors, including first-generation inhibitor midostaurin and second-generation inhibitors gilteritinib and quizartinib, have shown efficacy in treating *FLT3*-mutated AML. However, challenges such as resistance mechanisms and relapse necessitate further research and the development of combination strategies. Future directions include the exploration of novel agents, combination therapies, and a deeper understanding of the underlying biology of *FLT3*-mutated AML.

Table 2 provides an overview of *FLT3 *inhibitors and their associated clinical trials. Midostaurin, a multi-targeted kinase inhibitor, showed improved overall survival in combination with standard chemotherapy in a phase III trial (RATIFY) conducted in newly diagnosed *FLT3*-mutated AML patients [20]. Gilteritinib, a selective *FLT3* inhibitor, demonstrated superior overall survival compared to salvage chemotherapy in a phase III trial (ADMIRAL) involving relapsed/refractory *FLT3*-mutated AML patients [21]. Quizartinib, another selective *FLT3* inhibitor, was evaluated in a phase III trial (QuANTUM-First) to assess its efficacy compared to chemotherapy in newly diagnosed *FLT3*-ITD AML patients [25].

**Table 2:** FLT3 inhibitors and clinical trials. FLT3, Fms-like tyrosine kinase 3; ITD, internal tandem duplication; AML, acute myeloid leukemia

Inhibitor	Mechanism of action	Clinical trials
Midostaurin	Multi-targeted kinase inhibitor	Phase III trial (RATIFY) showed improved overall survival when combined with standard chemotherapy in newly diagnosed *FLT3*-mutated AML patients.
Gilteritinib	Selective *FLT3* inhibitor	Phase III trial (ADMIRAL) demonstrated superior overall survival compared to salvage chemotherapy in relapsed/refractory* FLT3*-mutated AML patients.
Quizartinib	Selective *FLT3* inhibitor	Phase III trial (QuANTUM-First) evaluated the efficacy of quizartinib compared to chemotherapy in newly diagnosed *FLT3*-ITD AML patients.

## Conclusions

Advancements in comprehending these mutations offer potential benefits for AML diagnosis and treatment. Molecular testing for *FLT3* mutations aids in accurate risk assessment and treatment guidance, with ongoing research focusing on overcoming treatment resistance and enhancing outcomes for *FLT3*-mutated AML. The identification of novel therapeutic targets and innovative strategies further expands the therapeutic landscape. Overall, the investigation of *FLT3* mutations in AML unveils key molecular mechanisms, impacting diagnosis, risk assessment, and treatment decisions. Persistent research efforts have the potential to enhance patient outcomes and foster more personalized therapies for *FLT3*-mutated AML.
